# Chemokine CXCL7 Heterodimers: Structural Insights, CXCR2 Receptor Function, and Glycosaminoglycan Interactions

**DOI:** 10.3390/ijms18040748

**Published:** 2017-04-01

**Authors:** Aaron J. Brown, Prem Raj B. Joseph, Kirti V. Sawant, Krishna Rajarathnam

**Affiliations:** Department of Biochemistry and Molecular Biology, and Sealy Center for Structural Biology and Molecular Biophysics, The University of Texas Medical Branch, Galveston, TX 77555, USA; aj3brown@utmb.edu (A.J.B.); pbjoseph@utmb.edu (P.R.B.J.); kisawant@utmb.edu (K.V.S.)

**Keywords:** chemokine, heterodimer, CXCL7, CXCR2, glycosaminoglycan, heparin, NMR

## Abstract

Chemokines mediate diverse fundamental biological processes, including combating infection. Multiple chemokines are expressed at the site of infection; thus chemokine synergy by heterodimer formation may play a role in determining function. Chemokine function involves interactions with G-protein-coupled receptors and sulfated glycosaminoglycans (GAG). However, very little is known regarding heterodimer structural features and receptor and GAG interactions. Solution nuclear magnetic resonance (NMR) and molecular dynamics characterization of platelet-derived chemokine CXCL7 heterodimerization with chemokines CXCL1, CXCL4, and CXCL8 indicated that packing interactions promote CXCL7-CXCL1 and CXCL7-CXCL4 heterodimers, and electrostatic repulsive interactions disfavor the CXCL7-CXCL8 heterodimer. As characterizing the native heterodimer is challenging due to interference from monomers and homodimers, we engineered a “trapped” disulfide-linked CXCL7-CXCL1 heterodimer. NMR and modeling studies indicated that GAG heparin binding to the heterodimer is distinctly different from the CXCL7 monomer and that the GAG-bound heterodimer is unlikely to bind the receptor. Interestingly, the trapped heterodimer was highly active in a Ca^2+^ release assay. These data collectively suggest that GAG interactions play a prominent role in determining heterodimer function in vivo. Further, this study provides proof-of-concept that the disulfide trapping strategy can serve as a valuable tool for characterizing the structural and functional features of a chemokine heterodimer.

## 1. Introduction

Chemokines, a large family of signaling proteins, mediate diverse biological processes, including innate and adaptive immunity, organogenesis, and tissue repair [1,2,3]. Common to these functions is the directed trafficking of various cell types through interactions with seven transmembrane G-protein coupled receptors. Chemokine–chemokine receptor interactions form an intricate network of crosstalk, with a given chemokine binding either a single or multiple receptors and a given receptor binding either a single or multiple chemokines [4,5]. Additional layers of complexity arise from chemokines existing in multiple states, from monomers, dimers, and tetramers to oligomers and polymers, and from their interactions with sulfated glycosaminoglycans (GAG) [6,7,8]. During active inflammation, local chemokine concentrations both in the free and GAG-bound forms could vary by orders of magnitude, which in turn could regulate the steepness and duration of chemotactic and haptotactic gradients [9,10]. Further, several lines of evidence indicate that chemokines also form heterodimers, suggesting yet another layer of complexity in regulating function.

Humans express ~50 different chemokines, which can be classified into subfamilies on the basis of the first two conserved cysteine residues as CXC, CC, CX_3_C, and XC [11,12]. Despite sequence identity that can be as low as 20%, chemokines share a similar structure at the monomer level. Considering that they are small (~8 to 10 kDa), they show a remarkable array of oligomeric states and binding interfaces. At the simplest level, chemokines share similar dimeric structures within a subfamily. The CXC-family forms globular dimers, with the first β-strand constituting the dimer interface, while the CC-family forms elongated dimers, with the N-loop constituting the dimer interface. However, this classification is not stringent, as some CC chemokines form CXC dimers [13] and some do not form dimers even at mM concentrations [14,15]. Chemokines that form tetramers exhibit both CXC and CC dimer interfaces [16,17,18]. Some chemokines form elongated polymers that have a completely different interface [19,20]. Further, lymphotactin, the only member of the C family, shows a typical chemokine fold and yet a completely different fold as a function of pH and solution conditions [21]. These properties speak to the inherent plasticity of the chemokine dimer interface. Considering that many chemokines are co-expressed under conditions of insult or co-exist in granules, it follows that chemokines are capable of forming heterodimers.

Functional studies have provided evidence for chemokine “synergy”, wherein the presence of multiple chemokines results in enhanced or altered activity [22,23,24,25]. Synergy is thought to play an important role at the onset of inflammatory signaling and has been attributed to altered receptor signaling [26] and/or heterodimer formation [27,28]. As an example, the functional potential of chemokine synergy was demonstrated in vivo using peptides that inhibit the CXCL4/CCL5 heterodimer in a mouse atherosclerosis model [29]. Many of the chemokines known to exist in platelets, as well as those that mediate neutrophil recruitment, form heterodimers. Several studies have provided evidence for heterodimers [30,31,32], but very little is known regarding the structural features, the molecular mechanisms underlying heterodimerization, or how heterodimers interact with their cognate receptors and GAGs. Such knowledge is essential to describe how the interplay between heterodimers, GAGs, and receptors mediates crosstalk between platelets and neutrophils towards the successful resolution of disease.

In this study, we investigated the molecular basis of heterodimer formation for the chemokine CXCL7 with chemokines CXCL1, CXCL4, and CXCL8. These chemokines co-exist in platelet granules, and their release upon platelet activation orchestrates neutrophils to the tissue injury site. CXCL7, CXCL1, and CXCL8, characterized by the conserved N-terminal “ELR” motif, direct neutrophil trafficking by activating the CXCR2 receptor [33]. CXCL4 is not a CXCR2 agonist as it lacks the ELR motif, but it plays an important role in promoting neutrophil adhesion [34]. We show that favorable packing and ionic interactions promote CXCL7-CXCL1 and CXCL7-CXCL4 heterodimers and that repulsive ionic interactions disfavor the CXCL7-CXCL8 heterodimer. Using a “trapped” disulfide-linked CXCL7-CXCL1 heterodimer, we provide definitive insights into GAG and receptor binding interactions. Interestingly, we observed that the trapped heterodimer was as active as the native proteins for CXCR2 function in a Ca^2+^ release assay. Further, GAG heparin interactions of the heterodimer are distinctly different from the CXCL7 monomer, and the GAG-bound heterodimer is unlikely to bind the receptor. Our observation that GAG binding interactions of a heterodimer could be quite different from the monomer suggests that these differences could play important roles in fine-tuning in vivo neutrophil recruitment and function. To our knowledge, this is the very first report of receptor and GAG interactions that could be unambiguously attributed to a chemokine heterodimer.

## 2. Results

### 2.1. NMR (Nuclear Magnetic Resonance) Characterization of CXCL7 Heterodimers

**Figure 1:**
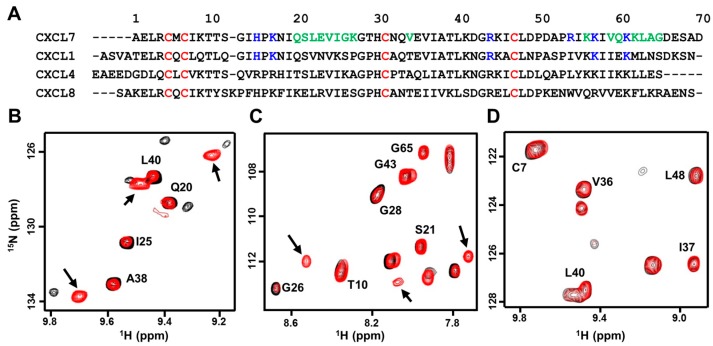
NMR (nuclear magnetic resonance) characterization of CXCL7 heterodimers. (**A**) Sequence alignment of platelet-derived CXC chemokines. GAG (glycosaminoglycans)binding residues identified from this study are in blue, dimer interface residues for CXCL7 are in green, and conserved Cys residues are in red; (**B**–**D**) Sections of the ^1^H–^15^N HSQC (heteronuclear single quantum coherence) spectra showing the overlay of CXCL7 in the free (black) and in the presence of CXCL1 (**B**, red), CXCL4 (**C**, red), and CXCL8 (**D**, red). Arrows indicate new peaks corresponding to the heterodimer. No new peaks were observed in the case of the CXCL8 titration.

CXCL7, compared to CXCL1 and CXCL8, forms weak dimers, and actually forms a tetramer at high concentrations. CXCL4, on the other hand, forms an even stronger tetramer. Tetramer structures of CXCL7 and CXCL4 reveal both CXC and CC type dimer interfaces [16,35]. CXC-type homodimers are stabilized by six H-bonds across the dimer interface β-strands and inter-subunit packing interactions between helical and β-sheet residues [16,17,36,37,38]. Sequences and the structures reveal that many of the hydrophobic dimer interface residues are conserved and that polar and charged dimer interface residues are not (Figure 1A). Comparing dimer interface residues of CXCL7 to CXCL1, CXCL4, and CXCL8 reveals ~40% to 60% similarity, suggesting that the propensity to form heterodimers could vary between each chemokine pair.

Solution nuclear magnetic resonance (NMR) spectroscopy is ideally suited for characterizing CXCL7 heterodimers compared to other techniques. We recently assigned the chemical shifts of the CXCL7 monomer and extensively characterized dimerization propensity as a function of solution conditions such as pH and buffer [39]. The heteronuclear single quantum coherence (HSQC) spectrum of ^15^N-labeled CXCL7 shows essentially a monomer along with some weak homodimer peaks. On titrating unlabeled CXCL1 or CXCL4 to ^15^N-CXCL7, the monomer and homodimer peaks gradually weaken, and new peaks appear that must correspond to the heterodimer (Figure 1B,C). These new peaks are in slow exchange with the CXCL7 monomer and homodimer. No changes were observed on titrating CXCL8 to ^15^N-CXCL7, indicating the absence of heterodimer formation (Figure 1D). We also carried out reverse titrations by titrating unlabeled CXCL7 to ^15^N-labeled CXCL1 or CXCL8. Titrating CXCL7 to ^15^N-CXCL1 resulted in the disappearance of monomer peaks and the appearance of new peaks confirming heterodimer formation. Conversely, titrating CXCL7 to ^15^N-CXCL8 resulted in no spectral changes.

**Figure 2:**
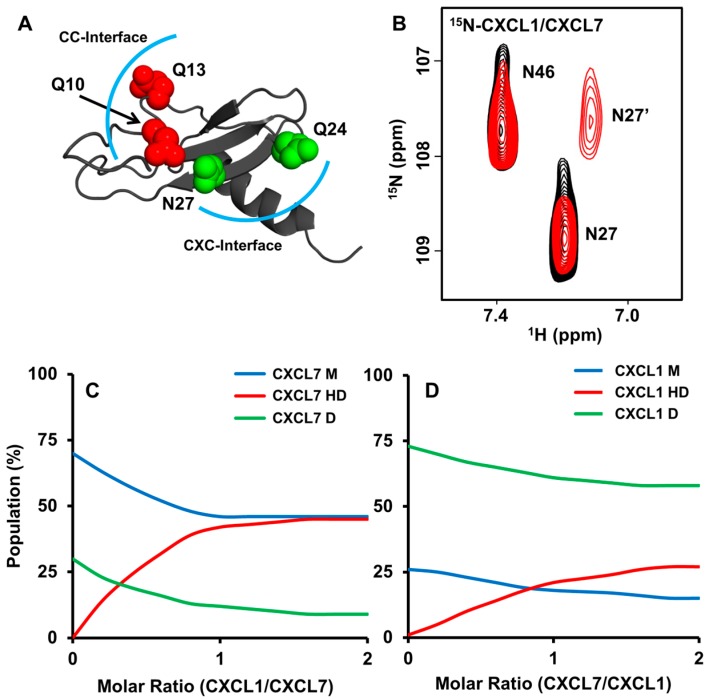
Characterization of the native CXCL7-CXCL1 heterodimer. (**A**) CXC (green) and CC (red) dimer-interface asparagine and glutamine residues are highlighted in the CXCL1 structure. The CXC and CC dimer interfaces are outlined with a blue arc; (**B**) Section of the spectra showing the CXCL1 side chain peaks for N27 and N46 in the free form (black) and in the presence of CXCL7 (red). Of the two peaks, only N27 shows reduced intensity and a new peak corresponding to the heterodimer (labeled as N27’); (**C**,**D**) Plots showing the relative populations of monomer (M), homodimer (D), and heterodimer (HD) based on NMR peak intensities during the course of the titration. Panel **C** shows the relative populations on adding CXCL1 to ^15^N-CXCL7, and panel **D** shows the relative populations on adding CXCL7 to ^15^N-CXCL1.

The appearance of a new peak during the course of a titration indicates that the environment of the particular residue in the heterodimer is different compared to the monomer or homodimer. On titrating CXCL1 to ^15^N-CXCL7, new peaks are observed that correspond to CXCL7 β_1_-strand residues S21, L22, V24, β_2_-strand residues V34, E35, V36, and I37, and C-terminal helical residues K56, K62, A64, and G65. These residues are located either at or proximal to the dimer interface. In the reverse titration of adding CXCL7 to ^15^N-CXCL1, new peaks corresponding to residues I23 to S30 of the dimer-interface β_1_-strand, T38 to L44 of the adjacent β_2_-strand, and C-terminal helical residues I58 to S69 are observed. These data collectively indicate that CXCL7-CXCL1 forms a CXC-type heterodimer and that the residues involved in packing interactions that stabilize the homodimers also stabilize the heterodimer. We also explored whether side chain chemical shifts of glutamine and asparagine can serve as probes for heterodimer formation. In CXCL1, a glutamine and an asparagine are located at the CXC dimer interface as well as a pair of glutamines in the CC dimer interface (Figure 2A). Upon titrating CXCL7, chemical shift changes were observed for CXC dimer-interface Q24 and N27 but not for CC dimer-interfaces Q10 and Q13 (Figure 2B), providing further structural evidence for a CXC-type dimer.

Peak intensities can provide valuable information on the relative populations of the monomer, homodimer, and heterodimer. We were able to track intensity changes for a number of residues upon titrating CXCL1 into ^15^N-CXCL7 and vice versa. During the course of the titration, populations of both the CXCL7 monomer and homodimer decrease and populations of the heterodimer increase. On adding excess CXCL1, heterodimer and monomer populations become comparable and the homodimer population becomes negligible (Figure 2C). However, in the case of CXCL1, the heterodimer population continues to increase, but the homodimer levels remain high and the monomer population becomes negligible (Figure 2D). The relative populations from both titrations indicate that the heterodimer is more favored than the CXCL7 homodimer but less favored than the CXCL1 homodimer.

We briefly describe our findings for the CXCL7-CXCL4 heterodimer. Considering that the tetramer structures of CXCL7 and CXCL4 reveal both CXC and CC dimer interfaces [16,17], heterodimerization could occur via one or both interfaces. However, similar to the CXCL7-CXCL1 heterodimer, most of the new peaks lie in proximity to the first and second β-strand residues and none were found close to the N-loop residues, indicating a CXC-type dimer interface. Side chain chemical shifts of Asn and Gln residues of the CXC dimer interface were also perturbed, providing further evidence for a CXC-type dimer.

### 2.2. Molecular Dynamics of Chemokine Heterodimers

We utilized a molecular dynamics-based approach to gain insight into the molecular basis for heterodimer formation. Energy minimized heterodimer structures were subjected to ~180 ns molecular dynamics (MD) simulations in order to arrive at a stable structure that had minimal fluctuations in backbone root-mean-square deviation (RMSD). To gain insight into the relative stabilities and better understand the structural features that mediate heterodimer formation, we examined several parameters during the course of the simulation; H-bonds and packing interactions of the dimer-interface residues, backbone φ–ψ angles, and charge-charge interactions. The MD simulations collectively indicated that a combination of favorable H-bonding, packing, and electrostatic interactions, similar to what drives any complex formation, dictate heterodimer formation.

**Figure 3:**
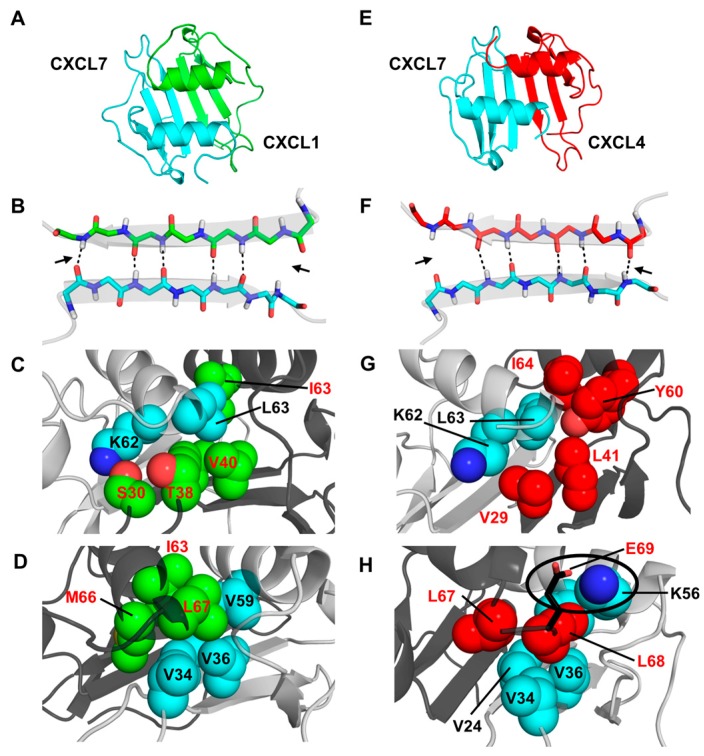
Structural features of the CXCL7 heterodimers. (**A**,**E**) Snap shots of the structural models of CXCL7-CXCL1 and CXCL7-CXCL4 heterodimers from the last 5 ns of the MD (molecular dynamics) simulations; (**B**,**F**) A schematic showing the β_1_-strand dimer interface H-bonds (dashed line) from the final 5 ns of the MD run. Arrows indicate transient H-bonds; (**C**,**D**) Packing interactions involving CXCL7 helical (cyan) and CXCL1 β-sheet residues (green) and CXCL1 helical (green) and CXCL7 β-sheet (cyan) residues; (**G**,**H**) Packing interactions involving CXCL7 helical (cyan) and CXCL4 β-sheet (red) residues and the CXCL4 helical (red) and CXCL7 β-sheet (cyan) residues. The circle highlights the potential ionic interaction between CXCL4 E69 and CXCL7 K56. Nitrogen atoms are shown in the conventional dark blue and oxygen in light red.

In the case of CXCL7-CXCL1, both monomer structures retained their tertiary fold. The H-bond network across the dimer interface β-strands remained intact for the CXCL7 residues L22 and V24 to CXCL1 residues V26 and V28, while peripheral H-bonds between CXCL7 G26 and Q20 to CXCL1 Q24 and S30 are transient throughout the run (Figure 3B). Backbone φ–ψ angles fall in the allowed region of the Ramachandran plot throughout the simulation. The dimer interface is stabilized by a number of favorable intermolecular packing interactions ? between M66 and L67 of CXCL1 and V24, G26, K56, and V59 of CXCL7 and between K62 and L63 of CXCL7 and V28, S30, V40, and I63 of CXCL1 (Figure 3C,D). Further, as is the case for the respective homodimer structures, the relative orientation of the helices remained parallel and in register (Figure 3A).

For the CXCL7-CXCL4 heterodimer, the final MD structure revealed that the monomer structures maintained their tertiary fold (Figure 3E). The dimer interface H-bonds across the β_1_-strands remain intact for CXCL7 residues L22 and V24 to CXCL4 residues L27 and V29, whereas the edge H-bonds (between CXCL7 Q20 and CXCL4 K31 and between CXCL7 G26 and CXCL4 T25) are transient (Figure 3F). The dimer is stabilized by favorable packing interactions between K62 and L63 of CXCL7 and V29, L41, Y60, and I64 of CXCL4 and between L67 and L68 of CXCL4 and V24, V34, V36, K56, V59, and L63 of CXCL7 (Figure 3G,H). Many of these residues are similar in the corresponding homodimers indicating conserved interactions (Figure 1A). However, there are unique structural differences in the heterodimer. For instance, E69 of CXCL4 (corresponding to A64 in CXCL7) is involved in ionic interactions with K56 from the opposite helix in CXCL7 (Figure 3H), and CXCL4 L67 and L68 are involved in additional packing interactions with CXCL7 L63 and V59. These new interactions result in the realignment of the helix and partial unwinding of the terminal helical residues.

For the CXCL7-CXCL8 heterodimer, despite favorable H-bonding and packing interactions, there was significant disruption of the tertiary fold due to unfavorable ionic interactions. The structure reveals that CXCL7 K27 and CXCL8 R68 are positioned across the dimer interface, resulting in electrostatic repulsion. In the CXCL8 homodimer, R68 is involved in favorable ionic interactions with E29 across the dimer interface. This swap from favorable to unfavorable interactions provides a molecular basis as to why CXCL7-CXCL8 fails to form a heterodimer.

### 2.3. Design and Characterization of a Trapped Heterodimer

Characterizing the structural and functional features of the native heterodimer is challenging due to contributions from two native homodimers and two native monomers. In principle, the solution contains as many as ten species; two monomers in the free and bound form, two dimers in the free and bound form, and heterodimers in the free and bound form. NMR experiments reduce this complexity by selectively labeling one of the monomers of the heterodimer, which simplifies the spectra to six species. In reality, we observe three sets of peaks due to fast exchange between the free and the bound form. Nevertheless, interpretation of such spectra is still challenging due to challenges in unambiguously assigning the chemical shifts of the newly formed heterodimer and tracking chemical shift perturbations (CSPs) of multiple species. This was evident when we initially attempted to characterize GAG binding to a wild type (WT) heterodimer mixture of ^15^N-CXCL7/CXCL1 or CXCL7/^15^N-CXCL1 at a 1:1 molar ratio. In order to overcome these limitations, we designed a disulfide-linked “trapped” CXCL7-CXCL1 heterodimer. 

**Figure 4:**
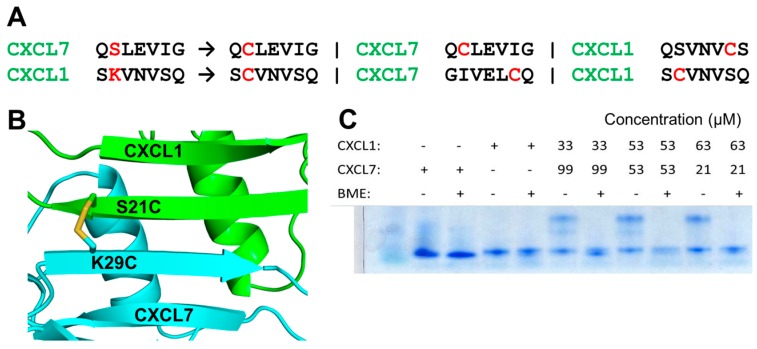
Characterization of the CXCL7-CXCL1 trapped heterodimer. (**A**) Trapping strategy showing cysteine mutations (in red) that will result only in a trapped heterodimer. Cysteines are too far away in the homodimer for disulfide formation; (**B**) A schematic of the heterodimer showing the location of the disulfide across the heterodimer interface and away from the two fold symmetry axis. CXCL7 is in cyan and CXCL1 is in green. (**C**) SDS-PAGE gel showing the formation of the disulfide bond. The higher molecular weight heterodimer band is observed only under non-reducing conditions. BME stands for β-mercaptoethanol.

**Figure 5:**
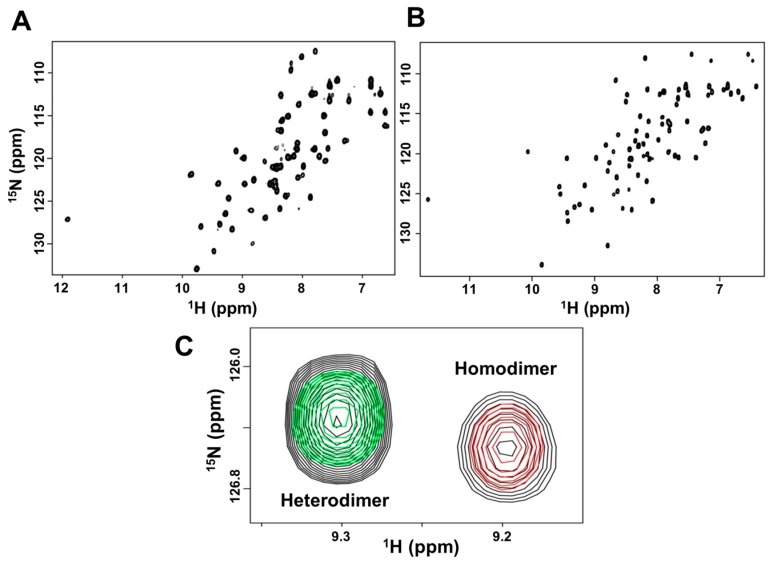
NMR structural features of the trapped heterodimer. ^1^H-^15^N HSQC spectra of the (**A**) ^15^N-CXCL7:CXCL1 and (**B**) ^15^N-CXCL1:CXCL7 trapped heterodimer. Spectra demonstrate a properly folded heterodimer with no evidence of monomer or homodimer; (**C**) The structure of the trapped heterodimer is similar to the native heterodimer. A section of the HSQC spectra of CXCL7 (red), trapped heterodimer (green), and a mixture of CXCL7 and CXCL1 in which both native heterodimer and native homodimer are present (black). Trapped heterodimer alone exists as a single species, free CXCL7 exists as monomers and homodimers, and native heterodimer is present along with native monomer and homodimer (Figure 2C). The trapped and native heterodimers have similar chemical shifts, as is evident from superimposed peaks. Please note the absence of a green peak superimposed on the homodimer peak. The peak corresponding to the monomer is not shown as it resonates outside of the displayed spectral window.

We used our heterodimer structural models from MD simulations to examine potential mutation sites in the CXCL7-CXCL1 heterodimer. To ensure formation of only the disulfide-linked heterodimer and no disulfide-linked homodimers, we looked for residues that are away from the two-fold symmetry axis. Other criteria that we considered were that these residues should minimally contribute to dimerization and/or influence the native fold. Our analysis pinpointed the solvent exposed β_1_-strand residues as likely candidates (Figure 4A,B). From this group, we chose the pair S21 from CXCL7 and K29 from CXCL1. The individual cysteine mutants (CXCL7 S21C and CXCL1 K29C) were recombinantly expressed and purified, and the trapped heterodimer was allowed to form by simple mixing of the proteins. We confirmed trapped heterodimer formation using SDS-PAGE, mass spectrometry, and NMR spectroscopy. Bands corresponding to the heterodimer were observed only under non-reducing conditions, indicating a disulfide-linked heterodimer (Figure 4C). The NMR spectra of the trapped heterodimer showed well-dispersed peaks characteristic of a single folded protein (Figure 5A,B). We also compared NMR spectra of the trapped heterodimer to the WT heterodimer (Figure 5C). The spectra were essentially similar except for residues in and around the mutation, indicating that the introduction of the disulfide does not perturb the native fold and that the trapped heterodimer retains the structural characteristics of the native heterodimer.

Knowledge of the chemical shifts is essential for NMR characterization of trapped heterodimer GAG interactions. Towards this, we carried out ^15^N-edited NOESY and TOCSY experiments on ^15^N-CXCL7-CXCL1 and ^15^N-CXCL1-CXCL7 trapped heterodimer samples. We were able to assign the backbone ^1^H and ^15^N chemical shifts of all CXCL1 residues and ~80% of CXCL7 residues. Some of the CXCL7 residues could not be assigned due to overlap or lack of sequential nuclear Overhauser effects (NOEs), but this was not limiting as most of the unassigned residues play no role in GAG interactions.

### 2.4. Heterodimer-GAG Interactions

**Figure 6:**
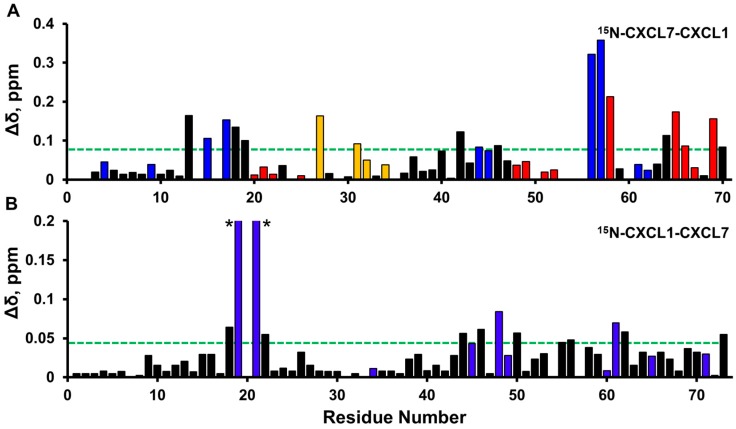
Histogram plots of chemical shift changes on heparin binding to trapped heterodimer. Heparin binding-induced chemical shift changes in CXCL7 (**A**) and CXCL1 (**B**) of the CXCL7-CXCL1 trapped heterodimer. Residues that show CSP above the threshold (dashed line) are considered involved in binding. Basic residues Arg, Lys, and His are shown in blue. CXCL7 residues that show sigmoidal binding profiles are shown in red, and CXCL7 residues showing normal hyperbolic profiles are shown in gold. Residues H19 and K21 (highlighted by *) show much higher CSPs (0.26 and 0.71 ppm, respectively).

We characterized the binding interactions of GAG heparin octasaccharide (dp8) by individual titrations to ^15^N-CXCL7-CXCL1 and ^15^N-CXCL1-CXCL7 trapped heterodimer samples. In the ^15^N-CXCL7-CXCL1 trapped heterodimer, significant perturbations were observed for N-loop, β_3_-strand, and α-helical residues. Of particular interest are the basic residues H15 and K17 of the N-loop, R44 and K45 from the β_3_-strand, and K56 and K57 from the helix (Figure 6A). CSPs for hydrophobic or acidic residues located proximal to these basic residues are likely due to indirect interactions. In the case of the ^15^N-CXCL1-CXCL7 trapped heterodimer, significant perturbations were observed for residues in the N-loop, β_3_-strand, and α-helix. These include the basic residues H19 and K21 of the N-loop, K45 and R48 of the 40s loop and β_3_-strand, and K61, K65, and K71 of the α-helix (Figure 6B).

**Figure 7:**
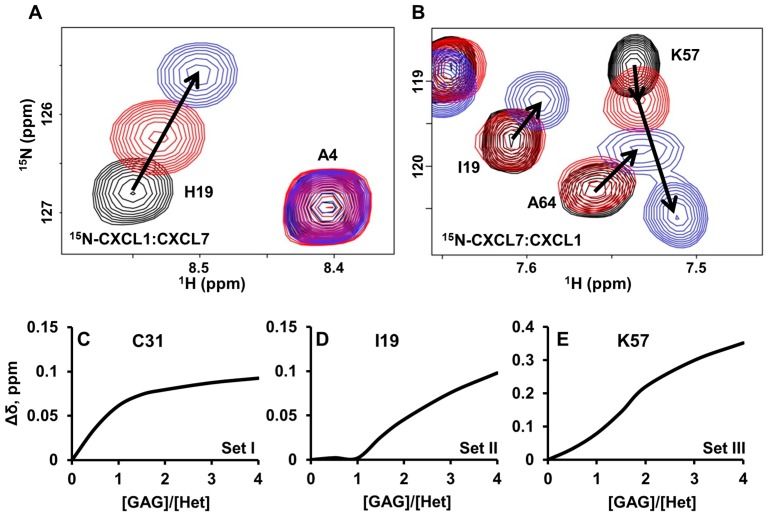
NMR characteristics of trapped heterodimer-heparin interactions. Sections of the ^1^H-^15^N HSQC spectra showing the overlay of CXCL7-CXCL1 trapped heterodimer in the free (black) and heparin dp8 bound form at 1:1 (red) and 1:4 (blue) molar ratios. Arrows indicate the direction of movement. (**A**) For CXCL1, only linear chemical shifts are observed; (**B**) In the case of CXCL7, both non-linear chemical shifts (K57) and delayed linear chemical shifts (I19 and A64) are observed; (**C**–**E**) Plots of binding-induced chemical shift changes on adding heparin. For CXCL7, (**C**) hyperbolic, (**D**) hyperbolic after a delay, and (**E**) sigmoidal profiles are observed.

Interestingly, the CSP profiles of CXCL1 versus CXCL7 residues were strikingly different (Figure 7A,B). Whereas all CXCL1 residues showed similar hyperbolic profiles, CXCL7 showed three distinctly different profiles. A subset of residues showed hyperbolic profiles (Figure 7C), a subset showed an initial delay in perturbation followed by a hyperbolic profile (Figure 7D), and a subset showed sigmoidal like profiles (Figure 7E). We define these residues as belonging to Set-I, Set-II, and Set-III, respectively. 

Set-I residues include K27 and C31 to V34 of the 30s-loop and K56 of the helix (Figure 6A). These residues lie along the dimer interface across from the CXCL1 β-sheet and the helical residues. Considering that these residues show hyperbolic perturbation profiles similar to CXCL1 residues, it is likely that their CSPs are due to indirect interactions of dp8 binding to CXCL1. For example, our structural model reveals that the CXCL7 K27 side chain is oriented towards the CXCL1 helix, likely making it sensitive to any structural changes in the CXCL1 helix, such as those often associated with dp8 binding.

Set-II residues include G13 to I19 of the N-loop, D42 to I46 of the β_3_-strand, and V59 to A64 of the helix (Figure 6A). These residues are located away from the dimer interface and are not influenced by CXCL1 binding. These perturbations can thus be attributed to direct dp8 binding to CXCL7.

Set-III residues include Q20 to I25 of the β_1_-strand, K57, I58, and G65 to A69 of the helix, and L48 to A52 that precede the helix (Figure 6A). In addition to sigmoidal binding profiles, these peaks showed non-linear chemical shift perturbations (Figure 7B). These residues are located at the crossroad between the CXCL7-GAG binding interface and the dimer interface. Therefore, their perturbations are likely a composite of both CXCL1 and CXCL7 dp8-binding. Residues K56, K57, and I58 are prominent examples. The K56 side chain is pointed towards the dimer interface, while K57 points out towards the N-loop. K56 shows a linear perturbation similar to CXCL1 residues, suggesting that its perturbation is due to direct or indirect interactions from dp8 binding to CXCL1. The initial perturbation of residues K57 and I58 can thus be attributed to a proximity effect of K56. However, the perturbation profile of K57 and I58 is altered upon further addition of dp8, indicating that these changes must be due to direct dp8 binding to CXCL7. Thus the sigmoidal profiles are a composite of CXCL1 and CXCL7 binding (Figure 7B,E). These data collectively indicate two independent binding sites, with one heparin binding one monomer and the second heparin binding the other monomer of the heterodimer, and that heparin first binds to CXCL1, due to higher affinity, and then to CXCL7.

As discussed above, characterizing GAG binding to the WT heterodimer is challenging. However, using the trapped heterodimer titration spectrum as a template, we explored whether we could characterize heparin binding to the native heterodimer. Indeed, we were able to track heparin binding to a few well-dispersed heterodimer peaks. For instance, upon titration, we observed a heterodimer peak, which showed significant CSP, a non-linear sigmoidal profile, and similar chemical shifts as K57 and I58 in the trapped heterodimer. Additionally, heterodimer peaks that could be assigned to Q20, L48, and G65 showed sigmoidal profiles similar to what was observed in the trapped heterodimer. These observations provide compelling evidence that binding interactions of the trapped heterodimer capture the complexity of the native heterodimer.

**Figure 8:**
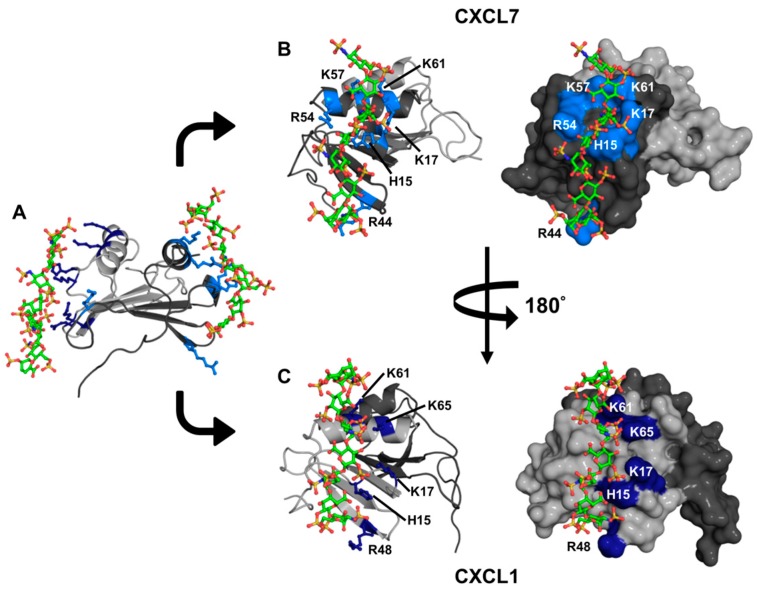
A model of the heparin-bound CXCL7-CXCL1 heterodimer complex. (**A**) Ribbon diagram showing that heparin binds to both monomers of the heterodimer. CXCL7 is shown in dark gray and CXCL1 light gray; (**B**,**C**) Cartoon and surface plots showing side views of the CXCL7 and CXCL1 monomer faces interacting with heparin dp8, respectively. The basic residues involved in binding are labeled and shown in blue.

To gain insight into the binding geometries, we generated models of the GAG heparin dp8 bound CXCL1-CXCL7 heterodimer complex using HADDOCK-based docking. We performed two independent runs. In run-I, restraints were given between one dp8 and CXCL7 and between another dp8 and CXCL1. In run-II, restraints were given between two GAGs and both monomers of the heterodimer. Both runs showed essentially the same binding geometry, with one GAG binding to each monomer of the heterodimer (Figure 8A). In CXCL7, the GAG-binding interface spans the β_3_-strand, the N-loop, and the helix and is mediated by H15 and K17 of the N-loop, R44 of the β_3_-strand, and R54, K57, and K61 of the helix (Figure 8B). In CXCL1, the GAG-binding interface also spans the β_3_-strand, the N-loop, and the helix and is mediated by H19 and K21 of the N-loop, R48 of the β_3_-strand, and K61 and K65 of the helix (Figure 8C). CXCL1 K45 and CXCL7 K27 were not involved in binding, though both showed significant CSP, indicating that their CSP is most likely due to indirect interactions. We also carried out modeling of one GAG to either CXCL1 or CXCL7 and observed the same binding interactions as observed for two GAGs. Our models provide the structural basis for stepwise and non-overlapping binding geometry, which is consistent with the NMR titrations. Further, the GAG-binding geometry is distinct from that observed in the CXCL1 dimer, wherein GAG binds across the β-sheet dimer interface [40]. Considering that previous studies have established that the N-loop residues in CXCL7 and CXCL1 are involved in receptor binding, the models also suggest that GAG-bound heterodimer cannot bind the receptor [39,41].

### 2.5. Heterodimer Receptor Binding Activity

**Figure 9:**
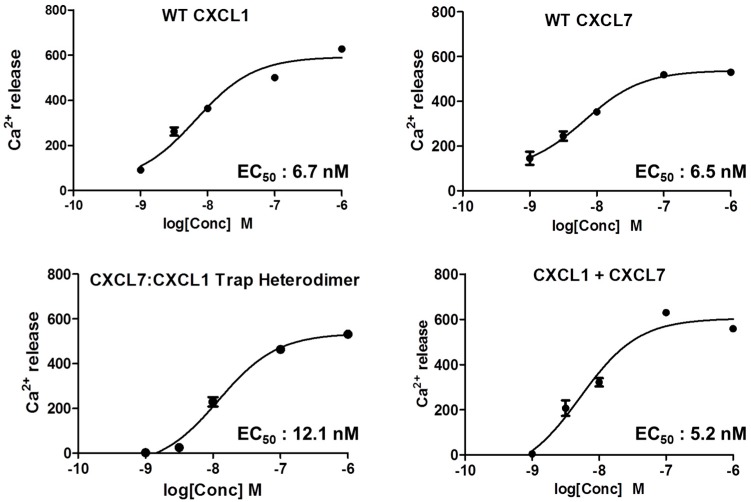
CXCR2 activity of the heterodimer. A plot showing the activity curves for WT CXCL1, WT CXCL7, 1:1 CXCL7/CXCL1 mixture, and the trapped CXCL7-CXCL1 heterodimer. The EC_50_ values indicate that the heterodimer binds and activates the receptor like the WT proteins.

We characterized receptor activity by measuring Ca^2+^ release using HL60 cells stably transfected with the CXCR2 receptor [41]. We compared the receptor activities of WT CXCL1, WT CXCL7, a mixture of both chemokines (CXCL7 and CXCL1), and our trapped heterodimer (CXCL7-CXCL1). The trapped heterodimer was as potent as the WT chemokines, and the activity of the mixture of CXCL1 and CXCL7 (that corresponds to the native heterodimer) was no different from the trapped heterodimer or WT proteins (Figure 9). These data indicate that there is no synergy and that essentially one of the monomers of the heterodimer binds and activates the receptor. Previous studies using a trapped homodimer for CXCL1 and CXCL8 have also shown that the activity of the homodimer was no different from the monomer [41,42,43].

## 3. Discussion

Animal model and in vitro studies have shown enhanced or altered activity for a wide variety of CXC, CC, and CXC/CC chemokine pairs [22,23,24,25,44,45,46]. For instance, high levels of CXCL1 (KC) and CXCL2 (MIP-2) have been observed in a number of murine disease models: virus-infected epithelial cells release multiple chemokines that direct neutrophil chemotaxis; peptides that inhibit CCL5/CXCL4 heterodimer formation alleviate atherosclerosis in a mouse model; and the CXCL7/CXCL4 pair compared to CXCL7 alone shows differential activity for neutrophil adhesion and transendothelial migration [29,47,48,49]. However, whether altered activity is due to non-additive receptor activity of two chemokines or to distinct heterodimer receptor activity is unknown.

Knowledge of the structural basis and molecular mechanisms by which chemokines form heterodimers is essential to understanding how heterodimers mediate function. In this study, using solution NMR spectroscopy, we were able to describe the structural features and molecular basis by which CXCL7 is able to form heterodimers with some chemokines but not with others. Further, using NMR spectroscopy, we were able to describe the molecular basis of heparin GAG binding to the CXCL7-CXCL1 heterodimer. NMR detects direct binding and does not require exogenous tagging, as do the fluorescence-based FRET/BRET methods, and so does not suffer from potential artifacts. Popular techniques for distinguishing between monomers and dimers such as gel filtration and native gel electrophoresis cannot distinguish between heterodimers and homodimers due to their similar size and molecular weight. Mass spectrometry and co-immunoprecipitation techniques have been used to detect chemokine heterodimers [30,50,51], but these techniques do not provide any insight into the molecular basis of heterodimer formation. NMR chemical shifts of the backbone amide (^1^H and ^15^N) are sensitive to secondary, tertiary, and quaternary structures. Therefore, under ideal conditions, NMR could distinguish heterodimers from homodimers and monomers. Previous NMR studies have shown heterodimer formation between CXCL4 and CXCL8 [32] and that the CCL2-CCL8 heterodimer is favored compared to the CCL2 homodimer [50] but did not describe the structural features of the heterodimer. This is challenging and requires chemical shift assignments not only of the monomer but also of the heterodimer.

The role of in vivo heterodimer function is dependent on receptor and GAG interactions. GAG interactions play multiple roles that include determining the makeup of the chemotactic/haptotactic gradients, influencing whether it is the free or GAG-bound chemokine that activates the receptor, and regulating the levels of the free monomer and homodimer. Further, free and GAG-bound heterodimer levels depend on the GAG affinities, the equilibrium constants (K_d_) of the heterodimer and of the two homodimers, and the relative amounts of the two chemokines. Using trapped dimers, it has been shown that the dimer could be as active as the monomer for CXCR2 function in cellular assays [41]. However, the in vivo recruitment activity of the monomers and dimers is distinctly different, indicating that the monomer-dimer equilibrium and GAG binding are coupled and regulate in vivo recruitment [8,52,53]. Therefore, any novel activity of the heterodimer can be inferred only under conditions in which the heterodimer dominates and in which its activity is different from monomers and dimers, and this becomes challenging if its levels are not high and/or its activity is not very different from monomers and homodimers.

In this study, using a disulfide-trapping strategy, we characterized heparin dp8 binding and CXCR2 activity of the CXCL7-CXCL1 heterodimer. We observed that the calcium release activity of the trapped CXCL7-CXCL1 heterodimer, which functions as a readout for the G-protein signaling pathway, was no different compared to the WT proteins. However, chemokine engagement of the CXCR2 receptor activates G-protein and β-arrestin signaling pathways and β-arrestin mediated endocytosis. Several studies have shown that a given chemokine-receptor pair, or multiple chemokines that target a single receptor, can have large differences in G-protein or β-arrestin mediated signaling or receptor internalization activities. Future functional studies of both G-protein and β-arrestin signaling and internalization activities are required to completely understand how heterodimers differ from monomers and homodimers in eliciting receptor function.

To our knowledge, this is the very first study that describes the GAG interactions and receptor activity of a heterodimer without interference from the monomers or homodimers. The GAG interactions of the heterodimer were strikingly different from the CXCL7 monomer and CXCL1 homodimer [39,40]. Further, a number of residues implicated in GAG binding also mediate receptor interactions, suggesting that a GAG-bound heterodimer is unlikely to activate the receptor. We conclude that differences in heterodimer-GAG interactions may play a role in fine-tuning chemotactic/haptotactic gradients and also control the amount of free chemokine available to activate the receptor. Finally, our strategy of engineering a disulfide-linked trapped chemokine heterodimer opens up new avenues to characterize in vivo heterodimer function and the role of differential receptor signaling pathways and to elucidate the heterodimer’s role for a variety of chemokine pairs in health and disease.

## 4. Materials and Methods

### 4.1. Molecular Dynamics Simulations

Initial structures were prepared using NMR or X-ray coordinates available from the protein data bank (PDB). The PDB IDs used were 1NAP (CXCL7) [16], 1MSG (CXCL1) [37], 1PFM (CXCL4) [35], and 1IL8 (CXCL8) [38]. Structures were generated by alignment of homodimer backbones and then removal of one of the monomers of each homodimer using PyMol [54]. In the heterodimer, the monomer structures were adjusted by translational and rotational motions about the two fold symmetry axis to align the hydrogen bond network across the β-strands of the dimer interface. The modelled heterodimer structures were then subjected to constrained energy minimization to eliminate any steric clashes, followed by free minimization using the AMBER 12 suite software and the ff03 force field [55,56]. The energy-minimized structures were subjected to an equilibration protocol in explicit solvent [57], followed by ~180 ns of MD production runs carried out using the PMEMD (Particle mesh Ewald molecular dynamics) module of the AMBER 12 software suite on the Lonestar Dell Linux Cluster at the Texas Advanced Computing Center (Texas Advanced Computing Center, The University of Texas, Austin, TX, USA). The trajectories were analyzed using AMBERtools 12, VMD, and PyMol [54,56,58].

### 4.2. Expression and Purification of Chemokines

Chemokines were expressed in *Escherichia coli* cultured in either LB or ^15^N-enriched minimal medium and purified using a combination of nickel column and reverse phase high-performance liquid chromatography, as previously described [59]. The CXCL7-CXCL1 trapped heterodimer was prepared by introducing a disulfide across the dimer interface. CXCL7 S21C and CXCL1 K29C mutants were purified using a Ni-NTA column, cleaved using Factor Xa, and were combined without further purification and left overnight at 35 °C. Heterodimer was purified using high performance liquid chromatography, lyophilized, and stored at −20 °C until further use.

### 4.3. NMR Spectroscopy

The samples were prepared in a 50 mM sodium phosphate buffer pH 7.4 at 25 °C containing 1 mM 2,2-dimethyl-2-silapentansesulfonic acid (DSS), 1 mM sodium azide, and 10% D_2_O. Heterodimer formation between two chemokines can be inferred from changes in the HSQC spectra on titrating an unlabeled chemokine to a ^15^N-labeled chemokine prepared in the same buffer. Initial ^15^N-labeled chemokine concentrations varied between 30 and 150 µM. The final molar ratios of labeled to unlabeled chemokine varied from 1:2 to 1:4. For these experiments, titrations were carried out until essentially no change in the spectra was observed. NMR experiments were performed on a Bruker Avance III 600 (with a QCI cryoprobe) or 800 MHz (with a TXI cryoprobe) spectrometer. All spectra were processed and analyzed using Bruker Topspin 3.2 or Sparky software [60]. 

The ^1^H and ^15^N chemical shifts of the trapped CXCL7-CXCL1 heterodimer were assigned using ^15^N-CXCL1-CXCL7 and ^15^N-CXCL7-CXCL1 samples prepared in 50 mM phosphate pH 6.0 and 35 °C. The concentrations of ^15^CXCL7-CXCL1 and CXCL7-^15^CXCL1 were 300 and 670 µM, respectively, and the assignments were obtained from analysis of ^1^H-^15^N heteronuclear NOESY and TOCSY experiments with mixing times of 150 and 80 ms, respectively.

### 4.4. Heparin-Heterodimer Interactions

The binding of heparin dp8 to the CXCL7-CXCL1 heterodimer was characterized using solution NMR spectroscopy in 50 mM phosphate buffer at pH 6.0 and 30 °C. The protein concentration for the titrations varied between 50 and 70 µM. Heparin dp8 was purchased from Iduron (Manchester, UK) and prepared in the same buffer (10 mM stock), and a series of ^1^H-^15^N HSQC spectra were collected upon titrating GAG until no changes in the spectra were observed. The final molar ratio of heterodimer to GAG was 1:4. For the trapped heterodimer, both ^15^N-CXCL7-CXCL1 and ^15^N-CXCL1-CXCL7 samples were used. For native heterodimer interactions, a mixture of CXCL7 and CXCL1 at 1:1 molar ratio was used. The final molar ratio of heterodimer to GAG was ~1:3 to 1:4. For all titrations, chemical shift perturbations were calculated as a weighted average of changes in the ^1^H and ^15^N chemical shifts, as described previously [61].

### 4.5. Heterodimer-GAG Docking

Molecular docking of heparin to the CXCL7-CXCL1 heterodimer was carried out using the High Ambiguity Driven biomolecular DOCKing (HADDOCK) approach, as described previously [62,63,64]. The CXCL7-CXCL1 heterodimer structure from MD studies and the NMR structure of heparin (PDB ID: 1HPN) [65] were used for docking. Ambiguous interaction restraints (AIRs) were selected based on NMR chemical shift perturbation data. The pair-wise “ligand interface RMSD matrix” over all structures was calculated and final structures were clustered using an RMSD cut-off value of 4 Å. The clusters were then prioritized using RMSD and a “HADDOCK score” (weighted sum of a combination of energy terms).

### 4.6. Receptor Activity of the Heterodimer

The CXCR2 receptor activity of the heterodimer was determined using a Ca^2+^ release assay, as described previously [41]. Ca^2+^ levels were measured using a FlexStation III microplate reader using the Calcium 5 assay kit (FLIPR, Molecular Devices). Differentiated HL60 cells expressing CXCR2 were incubated with varying concentrations of either WT CXCL1, WT CXCL7, a mixture of both WTs, or the trapped CXCL7-CXCL1 heterodimer. Changes in fluorescence of the Calcium 5 dye upon addition of chemokine were measured every 5 s for up to 500 s, and the agonist response was determined from the maximum change in fluorescence. EC_50_ values were calculated based on the response over a range of concentrations.

## Abbreviations

CXCL	CXC ligand
CXCR2	CXC chemokine receptor 2
CXCL7/NAP2	Neutrophil-activating peptide 2
GAG	Glycosaminoglycan
NAC	CXCR2 N-terminal domain
NMR	Nuclear Magnetic Resonance
HSQC	Heteronuclear single quantum coherence
CSP	Chemical shift perturbation
NOE	Nuclear Overhauser effect
AIR	Ambiguous interaction restraints
Dp8	Heparin octasaccharide
